# Photosynthesis, Chlorophyll Fluorescence, and Yield of Peanut in Response to Biochar Application

**DOI:** 10.3389/fpls.2021.650432

**Published:** 2021-05-31

**Authors:** Shujun Wang, Junlin Zheng, Yujia Wang, Qingfeng Yang, Taotao Chen, Yinglong Chen, Daocai Chi, Guimin Xia, Kadambot H.M. Siddique, Tieliang Wang

**Affiliations:** ^1^Key Laboratory of Agricultural Soil and Water Engineering of Liaoning Province, College of Water Conservancy, Shenyang Agricultural University, Shenyang, China; ^2^The UWA Institute of Agriculture, The University of Western Australia, Perth, WA, Australia; ^3^School of Agriculture and Environment, The University of Western Australia, Perth, WA, Australia

**Keywords:** biochar, chlorophyll fluorescence, plant nitrogen accumulation, photosynthetic traits, peanut yield

## Abstract

The effect of biochar application on photosynthetic traits and yield in peanut (*Arachis hypogaea* L.) is not well understood. A 2-year field experiment was conducted in Northwest Liaoning, China to evaluate the effect of biochar application [0, 10, 20, and 40 t ha^−1^ (B0, B10, B20, and B40)] on leaf gas exchange parameters, chlorophyll fluorescence parameters, and yield of peanut. B10 improved photochemical quenching at flowering and pod set and reduced non-photochemical quenching at pod set, relative to B0. B10 and B20 increased actual photochemical efficiency and decreased regulated energy dissipated at pod set, relative to B0. B10 significantly increased net photosynthetic rate, transpiration rate, stomatal conductance, and water use efficiency at flowering and pod set, relative to B0. Compared with B0, B10 significantly improved peanut yield (14.6 and 13.7%) and kernel yield (20.2 and 14.4%). Biochar application increased leaf nitrogen content. B10 and B20 significantly increased plant nitrogen accumulation, as compared to B0. The net photosynthetic rate of peanut leaves had a linear correlation with plant nitrogen accumulation and peanut yield. The application of 10 t ha^−1^ biochar produced the highest peanut yield by enhancing leaf photosynthetic capacity, and is thus a promising strategy for peanut production in Northwest Liaoning, China.

## Introduction

Peanut (*Arachis hypogaea* L.) is an annual legume crop. Global peanut consumption is increasing at a rate of around 3% per annum. China produces 40% of the world’s peanuts (FAOSTAT, 2018). Liaoning Province, is one of the main areas for peanut production in China and the primary export base of high-quality peanut. The Northwest Liaoning is a competitive producing area for peanut with a typical characteristic of sand and wind in semi-arid regions of Northeast China. However, peanut production in this area is limited by poor soil water and nutrient holding capacities, and water deficiency (Bai et al., 2014). Hence, the incorporation of plastic film mulching and supplemental irrigation have been studied as an extremely effective strategy with potential for decreasing soil evaporation, and enhancing crop growth, yield, and water use efficiency (Li and Gong, 2002; Ali et al., 2018; Xia et al., 2021a). However, the enhanced productivity under plastic mulches has been reported to result in lower soil fertility, which limit the subsequent crop productivity (Li et al., 2007; Steinmetz et al., 2016).

Biochar is produced through pyrolysis of biomass under limited oxygen environment (Lehmann et al., 2011). Generally, biochar with larger specific surface area, pore structure, abundant surface functional groups, and nutrient characteristics (e.g., C, N, P, K, S, Ca, and Mg) could improve soil sustainability (Ippolito et al., 2020; Leng et al., 2020; Ye et al., 2020). Most studies have shown that biochar is an effective agricultural practice for improving water and soil conditions in farmland and increasing crop yield and fertilizer use efficiency due to its unique structure (Clough et al., 2013; Laghari et al., 2015; Haider et al., 2017; Lin et al., 2017). The porous physical structure of biochar induces a sorption capacity to inorganic nitrogen and can potentially allow the slow release of nutrients to improve plant growth (Novak et al., 2012; El-Naggar et al., 2019). Biochar impacts the soil nitrogen, and is expected to enhance leaf nitrogen and photosynthesis (Kammann et al., 2011; Ali et al., 2020). Biochar addition to soil has positive effect on photosynthesis, being an important process that affects crop yield. When biochar application improves nitrogen accumulation, it also helps to increase leaf nitrogen content and therefore increases photosynthesis (Nguyen et al., 2017; Ali et al., 2020). The photosynthetic rate was increased at 40 t ha^−1^ biochar addition, and this enhancement in the leaf photosynthetic rate was due to the increased nitrogen accumulation (Ali et al., 2020). Biochar amendment increased the effective photochemical quantum yield of PSII and decreased the fluorescence yield for heat dissipation, therefore improved P_*n*_ (Abideen et al., 2020).

Most studies on peanut photosynthesis have focused on photosynthetic rate changes at different growth stages (Xu et al., 2015; Sun et al., 2018; Liu et al., 2019). However, limited information is available on the biochar effect on chlorophyll fluorescence and gas exchange parameters of peanut, especially *in situ* in the field. Aims of our study were to evaluate the effects of biochar application on photosynthesis from the perspective of chlorophyll fluorescence parameters, leaf nitrogen content, and plant nitrogen accumulation. We hypothesized that: (1) biochar application would improve peanut yield via enhancing leaf photosynthetic traits; (2) biochar improves the photosynthetic rate due to increasing the proportion of open photosystem II reaction centers, and nitrogen accumulation at low application rates.

## Materials and Methods

### Experimental Sites and Materials

**FIGURE 1:**
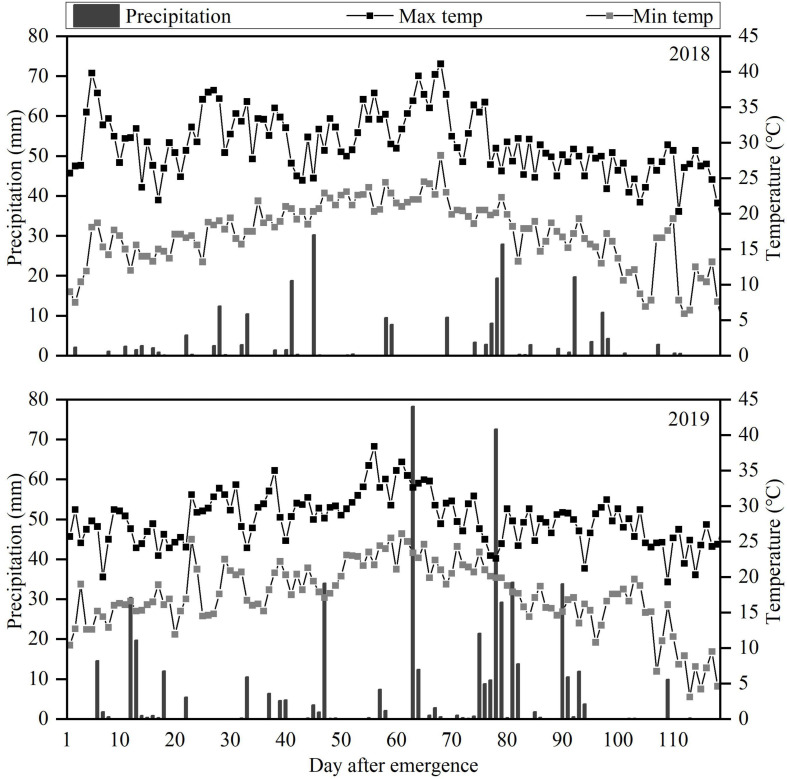
Daily precipitation and maximum (Max) and minimum (Min) temperatures during the 2018 and 2019 peanut growing seasons.

The field experiment was carried out at the Scientific Observation Experimental Station in Fuxin (42.11° N, 121.65° E), Liaoning Province, China, during the 2018 and 2019 growing seasons (May to October). This area with typical sand and wind conditions of semi-arid regions in Northeast China, has cold, dry winters and hot summers according to the Köppen-Geiger climate classification (Peel et al., 2007). Average annual rainfall is about 400 mm (60% from June to August), with average annual evaporation greater than 1,800 mm. Droughts are frequent. The daily weather data during the 2018 and 2019 peanut growing seasons are shown in Figure 1. The soil texture was sandy loam with pH 5.96, 1.44 g cm^–3^ bulk density, 19.5% (w/w) field capacity (FC), 0.62 g kg^−1^ total nitrogen, 142 mg kg^−1^ available potassium, and 18.1 mg kg^−1^ available phosphorus. The biochar was derived from maize straw pyrolyzed at 600°C with pH 8.14, 18.9%, carbon content, 0.58% nitrogen content, 4.76 g kg^−1^ available potassium, and 0.33 g kg^−1^ available phosphorus.

### Experimental Design, Establishment and Maintenance

The experiment was a randomized complete block design comprising four biochar application rates (0, 10, 20, and 40 t ha^−1^; B0, B10, B20, and B40) and three replicates (plots). Supplemental irrigation via a plastic mulched drip system was applied during the flowering and pod setting stages when peanut growth is more sensitive to water deficit than other stages. The field was irrigated up to 90% FC when the soil moisture content dropped to ≤55% FC. The biochar was fully mixed with the upper 0–20 cm soil layer by rotary before sowing in 2018. No additional biochar was applied in the second year. Basal fertilizers were applied at the rate of 50 kg ha^−1^ N, 170 kg ha^−1^ P_2_O_5_, and 156 kg ha^−1^ K_2_O.

Peanut cultivar Baisha 1016 origing in Guangdong Province was sown on 16 May 2018 and 19 May 2019 and harvested on 21 September 2018 and 23 September 2019. A trapezoidal ridge with a width of 0.7 m was formed by plough. Two rows were sown on the ridge of each hill (167,000 hills ha^−1^). The ridges were covered with white plastic film (0.008 mm thick) immediately after sowing. Each plot was 1 × 7.5 m^2^. Groundwater was used, with the irrigation amount determined by monitoring the volumetric water meter equipped in each plot. Other field management, including weeds, insects, and diseases control, were in line with local farmer practices.

### Sampling and Measurements

#### Chlorophyll Fluorescence Parameters

Chlorophyll fluorescence parameters of peanut were measured using the LI-6800 (LI-COR, Lincoln, NE, United States) photosynthesis measurement system with multiphase flash fluorescence (6800–01) at flowering (19 July 2018 and 16 July 2019) and pod set (8 August 2018 and 9 August 2019) on clear and cloudless days. To avoid influence of the changes in CO_2_ concentration in the air, the CO_2_ inlet of the instrument was connected to a CO_2_ cartridge (400 μmol mol^−1^). The third fully expanded leaf on the main stem were wrapped in aluminum foil. After remaining in complete darkness overnight, we measured minimal fluorescence yield (F_*o*_) using a measuring light (0.005 μmol m^–2^ s^−1^). Maximal fluorescence yield (F_*m*_) was measured using a 1 s saturating pulse at 8,000 μmol m^–2^ s^−1^ in dark-adapted leaves. The leaves were continuously illuminated for 20 min with an actinic light (1,400 μmol m^–2^ s^−1^) to record the steady-state yield of fluorescence (F_*s*_). Maximal light-adapted fluorescence yield (F_*m*_’) was determined by 8,000 μmol m^–2^ s^−1^. The actinic light was turned off, and minimal fluorescence yield (F_*o*_’) in light-adapted state was determined after 5 s of far-red illumination. The difference between the measured values of F_*m*_ and F_*o*_ is the variable fluorescence (F_*v*_). The chlorophyll fluorescence parameters were calculated using the following formulas (Kooten and Snel, 1990; Maxwell and Johnson, 2000; Kramer et al., 2004):

Fv/Fm=(Fm-Fo)/Fm

ΦPSII=(Fm-′Fs)/Fm′

ΦNPQ=Fs/Fm-′Fs/Fm

ΦNO=Fs/Fm

$$\text{qP}=\left({\text{Fm}}^{\prime }-\text{Fs}\right)/\left({\text{Fm}}^{\prime }-{\text{Fo}}^{\prime }\right)$$

$$\text{NPQ}=\text{Fm}/{\text{Fm}}^{\prime }-1$$

where F_*v*_/F_*m*_ is maximal photochemical efficiency of photosystem II (PSII), Φ_*PSII*_ is actual photochemical efficiency of PSII, Φ_*NPQ*_ is quantum yield for energy dissipated via Δ pH and xanthophyll-regulated processes, Φ_*NO*_ is quantum yield of non-regulated energy dissipated in PSII, and qP and NPQ are photochemical and non-photochemical quenching, respectively.

#### Gas Exchange Parameters, Leaf Nitrogen Content and Plant Nitrogen Accumulation

Gas exchange parameters were measured on the same dates and same leaves as those for chlorophyll fluorescence parameters measurements. Net photosynthesis rate (P_*n*_), transpiration rate (T_*r*_), stomatal conductance (G_*s*_), intercellular CO_2_ concentration (C_*i*_), and ambient CO_2_ concentration (C_*a*_) were measured with LI-6800 (LI-COR, Lincoln, NE, United States) photosynthesis measurement system. The stomatal limitation value (L_*s*_) was calculated as 1–C_*i*_/C_*a*_, and WUE was calculated as P_*n*_/T_*r*_ (Fang et al., 2018).

After the determination of gas exchange parameters, the third fully expanded leaf on the main stem of 20 plants in each pot was collected. Plant samples were collected at flowering and pod set, and were separated into various parts: roots, stems, leaves, and pods. All the samples were oven-dried at 105°C for 30 min and then at 80°C to constant weight. After weighing, these samples were ground into powder for measuring nitrogen content. The full-automatic KjelFlex K-360 analyzer (BUCHIK, Switzerland) was used to determine nitrogen content. Plant nitrogen accumulation was calculated by multiplying total nitrogen concentration in roots, stems, leaves, and pods with respective dry matter at flowering and pod set stages.

#### Yield and Yield Components

Peanuts were harvested from 1 m^2^ in the center of each plot. The pods were air-dried for about 1 week before being measured for peanut yield, kernel yield, 100-pod weight, and 100-kernel weight (Tan et al., 2018). The shelling percentage was calculated as (kernel weight/pod weight) × 100% (Luo et al., 2017).

### Statistical Analysis

SPSS 19.0 statistic software (SPSS Inc., Chicago, IL, United States) was used to perform the statistical analysis. Year and biochar application were assumed to be fixed factor and the replicates were assumed to be random factors. Error bars in the figures represent standard errors of the mean. Least significant differences were used to separate treatment means at the 5% probability level. Regression analysis was used to evaluate the relationships between leaf nitrogen content and net photosynthetic rate, net photosynthetic rate and peanut yield. The responses of chlorophyll fluorescence parameters, gas exchange parameters, leaf nitrogen content, yield, and yield components to biochar application were further analyzed with the principal component analysis in R studio version 1.1.442 using the Factoextra package (Kassambara, 2015).

## Results

### Chlorophyll Fluorescence Parameters

**FIGURE 2:**
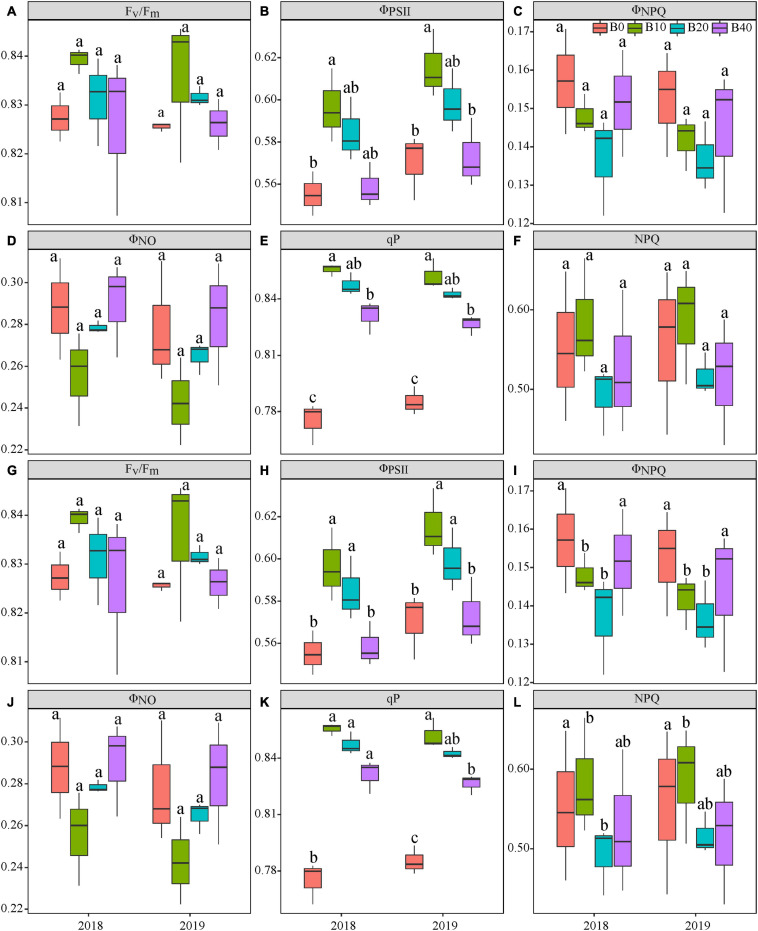
Chlorophyll fluorescence parameters at the flowering **(A–F)** and pod set **(G–L)** in peanut with four rates of biochar in the 2018 and 2019 growing seasons. F_*v*_/F_*m*_, maximal efficiency of PSII photochemistry after dark adaptation; Φ_*PSII*_, actual efficiency of PSII photochemistry after light adaptation; Φ_*NPQ*_, quantum yield for energy dissipated via Δ pH and xanthophyll-regulated processes; Φ_*NO*_, quantum yield of non-regulated energy loss in PSII; qP, photochemical quenching; and NPQ, non-photochemical quenching. B0, B10, B20, and B40 represent biochar application rates at 0, 10, 20, and 40 t ha^−1^, respectively. For each parameter in each year, mean data with different letters denote significant difference among treatments at *P* < 0.05.

Year, biochar application, and Y × B interaction had no significant effects on F_*v*_/F_*m*_ at flowering or pod set (Table 1 and Figures 2A,G). Biochar application had a significant effect on Φ_*PSII*_ at flowering and pod set, but there were no significant differences for year or Y × B interaction (Table 1). B10 increased Φ_*PSII*_ at flowering by 7.1 in 2018 and 8.8% in 2019, relative to B0 (Figure 2B). At pod set, B10 increased Φ_*PSII*_ by 13.0 in 2018 and 14.9% in 2019, and B20 increased Φ_*PSII*_ by 13.0 in 2018 and 12.8% in 2019, relative to B0 (Figure 2H). Among the four biochar treatments, B10 had the highest Φ_*PSII*_ values at flowering and pod set each year.

**TABLE 1:** Leaf chlorophyll fluorescence parameters and gas exchange parameters at the flowering and pod set in peanut with four rates of biochar in the 2018 and 2019 growing seasons.

		Flowering						Pod set					
		F_*v*_/F_*m*_	Φ_*PSII*_	Φ_*NPQ*_	Φ_*NO*_	qP	NPQ	F_*v*_/F_*m*_	Φ_*PSII*_	Φ_*NPQ*_	Φ_*NO*_	qP	NPQ
ANOVA	Y	ns	ns	ns	ns	ns	ns	ns	ns	ns	ns	ns	ns
	B	ns	**	ns	ns	**	ns	ns	**	**	ns	**	**
	Y × B	ns	ns	ns	ns	ns	ns	ns	ns	ns	ns	ns	ns

		**P_*n*_**	**T_*r*_**	**G_*s*_**	**L_*s*_**	**WUE**	**LNC**	**P_*n*_**	**T_*r*_**	**G_*s*_**	**L_*s*_**	**WUE**	**LNC**

ANOVA	Y	ns	ns	ns	ns	ns	ns	ns	ns	ns	ns	ns	ns
	B	**	**	**	**	**	**	ns	**	**	**	**	**
	Y × B	ns	ns	ns	ns	ns	ns	ns	ns	ns	ns	ns	ns

Y and B represent year and biochar application, respectively. F_*v*_/F_*m*_, maximal efficiency of PSII photochemistry after dark adaptation; Φ_*PSII*_, actual efficiency of PSII photochemistry after light adaptation; Φ_*NPQ*_, quantum yield for energy dissipated via Δ pH and xanthophyll-regulated processes; Φ_*NO*_, quantum yield of non-regulated energy loss in PSII; qP, photochemical quenching; NPQ, non-photochemical quenching. P_*n*_, net photosynthetic rate; T_*r*_, transpiration rate; G_*s*_, stomatal conductance; L_*s*_, stomatal limitation; WUE, water-use efficiency; and LNC, leaf nitrogen content. **denote significance at the 0.01 probability level. ns denotes non-significance.

Biochar application had a significant effect on Φ_*NPQ*_ at pod set but not at flowering (Table 1 and Figures 2C,I). Year and Y × B interaction had no significant effect on Φ_*NPQ*_ at flowering or pod set. There were no significant effects of biochar application, year, or Y × B interaction on Φ_*NO*_ at flowering or pod set (Table 1 and Figures 2D,J). At pod set, Φ_*NPQ*_ decreased with increasing biochar application rate to B10 and then increased. B10 and B20 decreased Φ_*NPQ*_ by 30.0 and 26.7% in 2018, and 27.6 and 24.1% in 2019, respectively, as compared to B0.

Biochar application had a significant effect on qP at flowering and pod set, but there were no significant differences for year or Y × B interaction (Table 1). At flowering, B10 enhanced qP by 11.7 and 7.6% in 2018 and 2019, respectively, relative to B0 (Figure 2E). At pod set, B10, B20, and B40 enhanced qP by 8.7, 7.2, and 5.8% in 2018, respectively, as compared to B0, but there were no significant differences between these treatments (Figure 2K). In 2019, B10 enhanced qP by 10.3%, relative to B0. Biochar application had a significant effect on NPQ at pod set but not at flowering. No significant differences were observed for year or Y × B interaction of NPQ at flowering or pod set (Table 1 and Figure 2F). At pod set, B10 and B20 decreased NPQ by up to 31.1% in 2018 and B10 decreased NPQ by 27.4% in 2019, as compared to B0 (Figure 2L).

### Gas Exchange Parameters and Leaf Nitrogen Content

**FIGURE 3:**
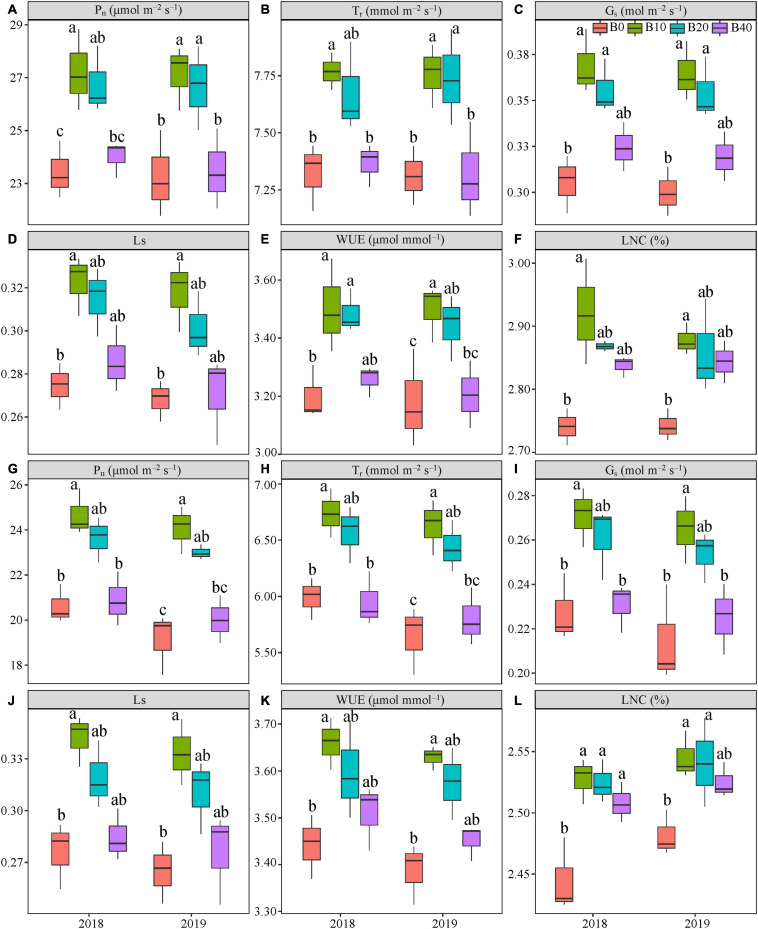
Gas exchange parameters at flowering **(A–F)** and pod set **(G–L)** in peanut applied with four biochar rates in 2018 and 2019 growing seasons. Y and B represent year and biochar application, respectively. P_*n*_, net photosynthetic rate; T_*r*_, transpiration rate; G_*s*_, stomatal conductance; L_*s*_, stomatal limitation; WUE, water-use efficiency; and LNC, leaf nitrogen content. B0, B10, B20, and B40 represent biochar application rates of 0, 10, 20, and 40 t ha^−1^, respectively. For each parameter in each year, mean data with different letters denote significant differences among treatments at *P* < 0.05.

Biochar application had a significant effect on P_*n*_ at flowering and pod set, but there were no significant effects for year or Y × B interaction (Table 1). B10 increased P_*n*_ at flowering by 16.1% in 2018, relative to B0 (Figure 3A). B10 and B20 increased P_*n*_ at flowering by up to 16.7% in 2019, as compared to B0. At pod set, B10 increased P_*n*_ by 19.6% in 2018 and 25.8% in 2019, relative to B0 (Figure 3G). B10 had the highest P_*n*_ at flowering and pod set in both years.

Application of biochar had significant effect on T_*r*_ at flowering and pod set. There were no significant effects of year or Y × B interaction on T_*r*_ during these two stages (Table 1). Compared with B0, B10 increased T_*r*_ at flowering by 6.1% in 2018. B10 and B20 increased T_*r*_ at flowering by up to 6.1% in 2019, relative to B0 (Figure 3B). B10 increased P_*n*_ at pod set by 12.5% in 2018 and 17.5% in 2019, relative to B0 (Figure 3H). Among the four biochar treatments, B10 had the highest T_*r*_ at flowering and pod set in both years.

The G_*s*_ was significantly affected by biochar application at both flowering and pod set stages. No significant differences in year or Y × B interaction of G_*s*_ were observed at both stages (Table 1). At flowering, B10 and B20 increased G_*s*_ by up to 20.8% in 2018 and 21.6% in 2019, relative to B0 (Figure 3C). B10 increased G_*s*_ at pod set by 19.2% in 2018 and 23.6% in 2019, as compared to B0 (Figure 3I). Among the four biochar treatments, B10 had the highest G_*s*_ at flowering and pod set in both years.

Application of biochar had a significant effect on L_*s*_ at flowering and pod set. There were no significant differences in year or Y × B interaction of L_*s*_ during these two stages (Table 1). Compared with B0, B10 increased L_*s*_ at flowering by 17.5% in 2018 and 18.6% in 2019, and at pod set by 23.8% in 2018 and 25.8% in 2019 (Figures 3D,J). The highest value of L_*s*_ at flowering and pod set in both years were appeared in B10.

Biochar application significantly affected WUE at flowering and pod set, but there were no significant effects for year or Y × B interaction (Table 1). At flowering, B10 and B20 increased WUE by up to 9.4% in 2018, as compared to B0 (Figure 3E). B10 increased WUE by 10.0% in 2019, as compared to B0. At pod set, B10 increased WUE by 6.3% in 2018 and 7.1% in 2019, relative to B0 (Figure 3K). B10 had the highest mean value of WUE at flowering and pod set in both years.

The LNC was significantly affected by biochar application at flowering and pod set. No significant differences in year or Y × B interaction were observed (Table 1). At flowering and pod set, with increasing biochar application rates, LNC increased to B10 and then decreased (Figures 3F,L). Among the four biochar treatments, B10 had the highest LNC at flowering and pod set in both years.

### Nitrogen Accumulation and Distribution

The effects of biochar application on root, stem, leaf, and total nitrogen accumulation were significant at flowering, but there were no significant effects for year or Y × B interaction (Table 2). B10 and B20 improved total nitrogen accumulation by 22.5 and 18.6% in 2018, 24.6 and 23.6% in 2019, relative to B0. Compare with B0, B10 and B20 improved root nitrogen accumulation by up to 25.6% in 2018 and 30.8% in 2019. The stem nitrogen accumulation in B10 improved by 21.6% in 2018, relative to B0. B10 and B20 improved stem nitrogen accumulation by up to 28.5% in 2019, as compared to B0. B10 improved leaf nitrogen accumulation by 26.4% in 2018 and 29.0% in 2019.

**TABLE 2:** Nitrogen accumulation and distribution at the flowering in peanut with four rates of biochar in the 2018 and 2019 growing seasons.

Year	Treatment	Total kg ha^−1^	Root		Stem		Leaf		Pod	
			kg ha^−1^	%	kg ha^−1^	%	kg ha^−1^	%	kg ha^−1^	%
2018	B0	64.9 ± 5.12b	2.89 ± 0.27c	4.47	18.5 ± 1.10b	28.5	36.7 ± 3.37b	56.5	6.84 ± 0.74a	10.5
	B10	79.5 ± 2.11a	3.63 ± 0.28a	4.57	22.5 ± 0.87a	28.4	46.4 ± 2.36a	58.4	6.86 ± 0.91a	8.65
	B20	77.0 ± 1.75a	3.61 ± 0.17a	4.69	21.9 ± 1.81ab	28.4	44.5 ± 1.64ab	57.8	6.99 ± 0.73a	9.09
	B40	70.4 ± 2.94ab	2.93 ± 0.20b	4.16	20.7 ± 1.12ab	29.4	39.7 ± 1.16ab	56.5	7.00 ± 0.92a	9.93
2019	B0	61.9 ± 2.54b	2.73 ± 0.26b	4.43	17.2 ± 0.90b	27.8	35.2 ± 1.56c	56.9	6.78 ± 0.31a	11.0
	B10	77.1 ± 6.54a	3.51 ± 0.34a	4.56	21.4 ± 1.56a	27.8	45.4 ± 5.39a	58.8	6.81 ± 0.64a	8.90
	B20	76.5 ± 3.88a	3.57 ± 0.48a	4.68	22.1 ± 1.64a	28.9	44.0 ± 4.02ab	57.5	6.82 ± 0.70a	8.95
	B40	69.4 ± 1.05ab	3.02 ± 0.27b	4.35	19.2 ± 1.80ab	27.6	40.4 ± 1.44abc	58.2	6.81 ± 0.35a	9.82
ANOVA										
Y		ns	ns		ns		ns		ns	
B		**	**		**		**		ns	
Y × B		ns	ns		ns		ns		ns	

Y and B represent year and biochar application, respectively. B0, B10, B20, and B40 represent biochar application rates at 0, 10, 20, and 40 t ha^−1^, respectively. For each parameter in each year, mean data with different letters denote significant difference among treatments at *P* < 0.05. **denote significance at the 0.01 probability level. ns denotes non-significance.

The root, stem, leaf, pod and total nitrogen accumulation were significantly affected by biochar application at pod set. No significant differences in year or Y × B interaction of root, stem, leaf, pod, and total nitrogen accumulation were observed at pod set (Table 3). The total nitrogen accumulation for B10 and B20 were higher than that of B0 by 25.0 and 15.3% in 2018, 23.7 and 20.3% in 2019, respectively, as compared to B0. B10 improved root nitrogen accumulation by 30.4% in 2018, relative to B0. Compared with B0, B10, and B20 improved root nitrogen accumulation by up to 30.0% in 2019. The stem nitrogen accumulation for B10 was 21.0% in 2018 and 17.8% in 2019 higher than that of B0. B10 and B20 improved leaf nitrogen accumulation by up to 24.0% in 2018 and 24.3% in 2019, relative to B0. Compared with B0, B10 improved pod nitrogen accumulation 27.0% in 2018. B10 and B20 improved pod nitrogen accumulation by 26.3% and 20.6% in 2019, as compared to B0.

**TABLE 3:** Nitrogen accumulation and distribution at the pod set in peanut with four rates of biochar in the 2018 and 2019 growing seasons.

Year	Treatment	Total kg ha^−1^	Root		Stem		Leaf		Pod	
			kg ha^−1^	%	kg ha^−1^	%	kg ha^−1^	%	kg ha^−1^	%
2018	B0	124 ± 2.8b	3.16 ± 0.28b	2.54	27.1 ± 1.53b	21.8	36.6 ± 1.72b	29.4	57.5 ± 0.92b	46.2
	B10	155 ± 7.6a	4.12 ± 0.25a	2.66	32.8 ± 1.04a	21.1	45.4 ± 2.40a	29.3	73.0 ± 4.75a	47.0
	B20	143 ± 8.3a	3.89 ± 0.18ab	2.73	30.8 ± 1.25ab	21.6	44.0 ± 2.57a	30.8	64.2 ± 5.87ab	44.8
	B40	130 ± 2.0b	3.35 ± 0.19b	2.58	28.4 ± 1.18b	21.9	38.4 ± 2.10b	29.6	59.5 ± 3.15b	45.9
2019	B0	118 ± 6.0b	3.00 ± 0.18b	2.55	25.9 ± 0.83b	22.0	35.0 ± 2.96b	29.7	54.0 ± 3.68b	45.8
	B10	146 ± 2.2a	3.90 ± 0.26a	2.67	30.5 ± 1.87a	20.9	43.5 ± 1.17a	29.8	68.2 ± 2.65a	46.7
	B20	142 ± 8.4a	3.75 ± 0.28a	2.65	30.0 ± 2.07ab	21.1	43.2 ± 2.78a	30.4	65.1 ± 3.79a	45.8
	B40	129 ± 3.5b	3.46 ± 0.10ab	2.69	28.0 ± 1.90ab	21.8	38.8 ± 3.70ab	30.0	58.7 ± 1.28ab	45.6
ANOVA										
Y		ns	ns		ns		ns		ns	
B		**	**		**		**		**	
Y × B		ns	ns		ns		ns		ns	

*Y and B represent year and biochar application, respectively. B0, B10, B20, and B40 represent biochar application rates at 0, 10, 20, and 40 t ha^−1^, respectively. For each parameter in each year, mean data with different letters denote significant difference among treatments at *P* < 0.05. ^∗∗^ and ^∗^ denote significance at the 0.01 and 0.05 probability levels, respectively. ns denotes non-significance.*

### Yield and Yield Components

Peanut yield, kernel yield and shelling percentage were significantly affected by year and biochar application (Table 4). There was a significant Y × B interaction for shelling percentage, but not for peanut yield or kernel yield. B10 and B20 increased peanut yield by 14.6 and 10.7% in 2018, 13.7 and 11.3% in 2019, respectively, relative to B0. B10 increased kernel yield by 20.2% in 2018 and 14.4% in 2019, relative to B0. B10 and B20 had similar shelling percentages to B0, while B40 had 4.4% lower shelling percentage than B0. Among the four biochar treatments, B10 had the highest peanut yield in both years. No significant differences occurred between years, biochar application, or Y × B interaction for 100-pod weight or 100-kernel weight.

**TABLE 4:** Yield and yield components of peanut applied with four rates of biochar in the 2018 and 2019 growing seasons.

Year	Treatment	Yield (t ha^−1^)	Kernel yield (t ha^−1^)	100-pod weight (g)	100-kernel weight (g)	Shelling percentage (%)
2018	B0	5.68 ± 0.17b	3.91 ± 0.14b	198 ± 7.8a	86 ± 3.0a	68.8 ± 0.91b
	B10	6.51 ± 0.14a	4.70 ± 0.13a	200 ± 6.4a	87 ± 2.7a	72.3 ± 0.62a
	B20	6.29 ± 0.19a	4.30 ± 0.17ab	196 ± 3.7a	86 ± 2.7a	68.4 ± 0.58b
	B40	5.69 ± 0.18b	3.95 ± 0.15b	194 ± 8.1a	85 ± 1.5a	69.4 ± 0.72b
2019	B0	5.48 ± 0.22b	3.76 ± 0.15bc	187 ± 5.2a	78 ± 2.4a	68.6 ± 0.35ab
	B10	6.23 ± 0.10a	4.30 ± 0.13a	190 ± 10.1a	80 ± 2.8a	69.1 ± 1.00a
	B20	6.10 ± 0.10a	4.06 ± 0.11ab	190 ± 10.6a	82 ± 3.7a	66.6 ± 0.92bc
	B40	5.49 ± 0.17b	3.60 ± 0.12c	179 ± 7.5a	77 ± 5.6a	65.6 ± 0.65c
ANOVA	Y	*	**	ns	ns	**
	B	**	**	ns	ns	**
	Y × B	ns	ns	ns	ns	*

*Y and B represent year and biochar application, respectively. B0, B10, B20, and B40 represent biochar application rates of 0, 10, 20, and 40 t ha^−1^, respectively. For each parameter in each year, mean data with different letters denote significant differences among treatments at *P* < 0.05. ^∗∗^ and ^∗^ denote significance at the 0.01 and 0.05 probability levels, respectively. ns denotes non-significance.*

### Relationship Between Net Photosynthetic Rate, Leaf Nitrogen Content, and Peanut Yield

**FIGURE 4:**
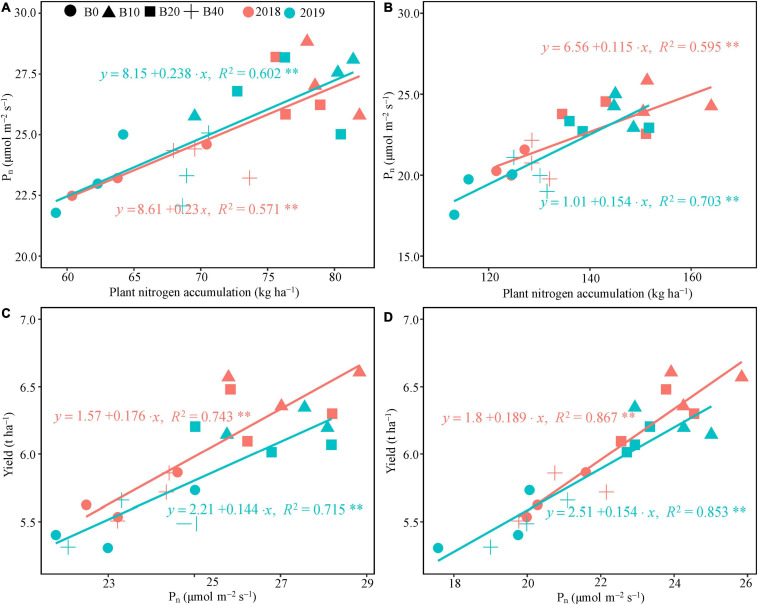
Relationship between plant nitrogen accumulation and photosynthetic rate, and yield and photosynthetic rate at flowering **(A,C)** and pod set **(B,D)** in peanut applied with four rates of biochar in the 2018 and 2019 growing seasons. B0, B10, B20, and B40 represent biochar application rates at 0, 10, 20, and 40 t ha^−1^, respectively. P_*n*_, net photosynthetic rate; **represents significant correlations at the *P* < 0.01 level.

The regression analysis indicated that P_*n*_ had a significant linear correlation with plant nitrogen accumulation at flowering and pod set in 2018 and 2019 (Figures 4A,B). Plant nitrogen accumulation explained 57.1 and 59.5% of the variation in P_*n*_ at flowering and pod set in 2018, and 60.2 and 70.3% in 2019, respectively. Positive correlations occurred between P_*n*_ at flower and pod set and peanut yield in both years (Figures 4C,D), explaining 74.3 and 86.7% of the variation in peanut yield in 2018, and 71.5 and 85.3% in 2019, respectively.

### PCA Analysis for Yield and Photosynthetic Traits of Peanut

**FIGURE 5:**
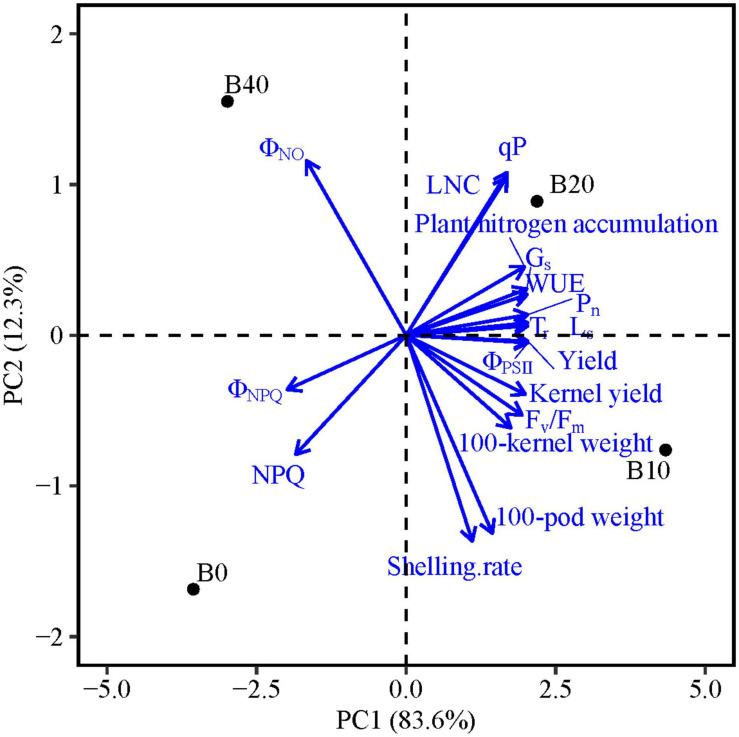
Principal component analyses of chlorophyll fluorescence parameters, gas exchange parameters, leaf nitrogen content, yield, and yield components of peanut in response to four biochar application rates. Means for flowering and pod set are for 2 years. F_*v*_/F_*m*_, maximal efficiency of PSII photochemistry after dark adaptation; Φ_*PSII*_, actual efficiency of PSII photochemistry after light adaptation; Φ_*NPQ*_, quantum yield for energy dissipated via Δ pH and xanthophyll-regulated processes; Φ_*NO*_, quantum yield of non-regulated energy loss in PSII; qP, photochemical quenching; NPQ, non-photochemical quenching; P_*n*_, net photosynthetic rate; T_*r*_, transpiration rate; G_*s*_, stomatal conductance; L_*s*_, stomatal limitation; WUE, water use efficiency; and LNC, leaf nitrogen content; B0, B10, B20, and B40 represent biochar application rates of 0, 10, 20, and 40 t ha^−1^, respectively.

The PCA results show that PC1 and PC2 explain 95.9% of the variation in functional traits (Table 5). PC1 explains 83.6% of the variability, and accounted mainly for chlorophyll fluorescence parameters (F_*v*_/F_*m*_, Φ_*PSII*_, Φ_*NPQ*_, Φ_*NO*_, qP, and NPQ), gas exchange parameters (P_*n*_, T_*r*_, G_*s*_, L_*s*_, and WUE), LNC, plant nitrogen accumulation and yield and yield components (kernel yield, 100-pod weight, and 100-kernel weight; Figure 5). PC2 explains 12.3% of the variability and accounts for shelling percentage. The loadings for qP, Φ_*PSII*_, gas exchange parameters, LNC, plant nitrogen accumulation, yield, and yield components are in quadrant I and IV, and Φ_*NPQ*_, Φ_*NO*_, and NPQ are in quadrants II and III, and Φ_*NPQ*_, Φ_*NO*_, and NPQ represent limitations in photosynthetic capacity. Φ_*PSII*_, Φ_*NPQ*_, and Φ_*NO*_ are distributed in different quadrants, indicating compensation effects of photochemical efficiency for dissipation by regulated and non-regulated energy losses. B10 and B20 are located in quadrants I and IV, which have a significant effect on peanut productivity, while B40 and B0 are in quadrant II and III, where absorbed light energy is lost by heat dissipation. The loading arrow of B10 is longer than that of B20. Thus, B10 in quadrant IV is an appropriate biochar application rate for relatively high photosynthetic capacity and peanut productivity.

**TABLE 5:** Variable loading scores of 18 parameters for four biochar application rate and the proportion of variation of each principal component.

Traits	PC1	PC2
*Chlorophyll fluorescence parameters*		
F_*v*_/F_*m*_	**0.95**	−0.26
Φ_*PSII*_	**0.99**	−0.02
Φ_*NPQ*_	−**0.97**	−0.17
Φ_*NO*_	−**0.81**	0.56
qP	**0.82**	0.52
NPQ	−**0.90**	−0.38
**Gas exchange parameters, leaf nitrogen content and plant nitrogen accumulation**		
P_*n*_	**0.99**	0.06
T_*r*_	**0.99**	0.03
G_*s*_	**0.98**	0.15
L_*s*_	**0.99**	0.04
WUE	**0.99**	0.13
Leaf nitrogen content	**0.82**	0.51
Plant nitrogen accumulation	**0.97**	0.22
*Yield and yield components*		
Yield	**0.99**	−0.01
Kernel yield	**0.97**	−0.19
100-pod weight	**0.70**	−0.64
100-kernel weight	**0.86**	−0.30
Shelling percentage	0.54	−**0.66**
Eigenvalue	15.1	2.2
Variance (%)	83.6	12.3
Cumulative variance (%)	83.6	95.9

*F_*v*_/F_*m*_, maximal efficiency of PSII photochemistry after dark adaptation; Φ_*PSII*_, actual efficiency of PSII photochemistry after light adaptation; Φ_*NPQ*_, quantum yield for energy dissipated via Δ pH and xanthophyll-regulated processes; Φ_*NO*_, The quantum yield of non-regulated energy loss in PSII; qP, photochemical quenching; NPQ, non-photochemical quenching; P_*n*_, net photosynthetic rate; T_*r*_, transpiration rate; G_*s*_, stomatal conductance; L_*s*_, stomatal limitation; and WUE, water use efficiency. For each parameter, the largest variable loading scores in the two components are in bold.*

## Discussion

### Effect of Biochar on Gas Exchange Parameters of Peanut

Peanut is a C3 crop with high potential for photosynthetic capacity. Therefore, exploring the photosynthetic capacity of peanut is an effective method for improving its productivity (Zelitch, 1982). Some studies have shown that biochar might improve the photosynthetic capacity of crop leaves (Rehman et al., 2016, 2019; Abbas et al., 2017). Biochar application improved leaf photosynthetic rate, which was due to the amelioration of soil physicochemical properties that ultimately increased nitrogen accumulation, and consequently enhanced photosynthetic rate (Liu et al., 2018; Huang et al., 2019; He et al., 2020). In our study, B10 enhanced P_*n*_, T_*r*_, G_*s*_, L_*s*_, and WUE at critical periods of peanut growth. In contrast, 40 t ha^−1^ biochar decreased these parameters, relative to 10 t ha^−1^ biochar (Figure 3), indicating that more biochar is not always beneficial for leaf photosynthesis. Differences in P_*n*_ among the four biochar rates could be due to the positive correlation between nitrogen accumulation and P_*n*_ at flowering and pod set in both years, as biochar application increased plant nitrogen accumulation. Plant nitrogen accumulation was higher in B10 and B20, but decreased in B40 at flowering and pod set (Tables 2, 3). The highest rate of biochar (40 t ha^−1^) may limit plant nitrogen accumulation which decreased leaf photosynthesis, which was likely attributed to nitrogen immobilization caused by the high C/N ratio (Asai et al., 2009). Photosynthetic rate had a positive relationship with LNC (Evans, 1989). In this study, B10 improved LNC at flowering (vegetative growth) by up to 6.6%. The significant increase in LNC at pod set was modest and may be due to reduction at pod set (reproductive growth), with more nitrogen transformed to pod (Tables 2, 3). Furthermore, the increase in dry matter production may have decreased leaf nitrogen due to the dilution effect (Guo et al., 2021). This enhancement of leaf photosynthetic rate could be explained by increased G_*s*_ and T_*r*_ after biochar application (Figure 3). The improving G_*s*_ and T_*r*_ may be associated with the increased soil water holding capacity, which might be resulting from the porous physical structure of biochar (Laghari et al., 2015; He et al., 2020). Additionally, some evidences suggested that biochar benefited root morphological development, including increased root volume, surface area and root density, to acquire more nutrients and water for enhancing photosynthesis (Bruun et al., 2014; Xiang et al., 2017). In fact, our study observed that 10 t ha^−1^ biochar promoted root morphology of peanut (Xia et al., 2021b). Hence, 10 t ha^−1^ biochar improved the nitrogen accumulation and photosynthetic rate, and consequently peanut yield.

### Effect of Biochar on Chlorophyll Fluorescence Parameters of Peanut

Chlorophyll fluorescence is an important photosynthetic parameter that reflects the absorption and utilization of light energy in PSII. F_*v*_/F_*m*_ represents the conversion efficiency of primary light energy in the PSII reaction center. Decreases in F_*v*_/F_*m*_ are often observed when plants are exposed to abiotic and biotic stresses in the light (Baker, 2008). In our study, F_*v*_/F_*m*_ did not significantly differ between treatments at flowering and pod set in either year (Figures 2A,G), which is consistent with Marks et al. (2016). Φ_*PSII*_ is an indicator of the electron transport rate in leaves, and higher Φ_*PSII*_ indicates a higher capacity of leaves to convert photon energy into chemical energy (Li et al., 2010). Φ_*NPQ*_ is an important indicator of photo-protection energy dissipation, and higher Φ_*NPQ*_ value shows a higher capacity to eliminate redundancy light energy by regulatory heat dissipation mechanism. Φ_*NO*_ is the combined pathway of radiative and non-radiative deexcitation reactions, and higher Φ_*NO*_ indicates that the absorbed light energy cannot be consumed completely through photochemical energy conversion and protective regulation mechanisms (Kramer et al., 2004; Klughammer and Schreiber, 2008; Chen et al., 2017). In this study, no significant difference in Φ_*NO*_ occurred between treatments at flowering or pod set in either year (Figures 2D,J). In terms of energy distribution, B10 promoted photosynthetic activity in peanut leaves, significantly increasing Φ_*PSII*_, and decreasing Φ_*NPQ*_ and Φ_*NO*_ at flowering and pod set in both years (Figure 2). B0 and B40 decreased Φ_*PSII*_ and increased Φ_*NPQ*_, indicating that an increase in regulated heat dissipation could protect the photosynthetic apparatus. qP represents the proportion of open PSII reaction centers (Hazrati et al., 2016). NPQ mainly comprises regulated and non-regulated energy dissipation and indicates that the light energy absorbed by PSII antenna pigments cannot be used for photochemical electron transfer, which dissipates as heat (Long et al., 2013; Perkins et al., 2018). Tang et al. (2020) reported that biochar pyrolyzed at 600°C increased qP and decreased NPQ, relative to the no-biochar treatment. Our results showed that B10 and B20 improved qP at flowering and pod set, and reduced NPQ at pod set in both years (Figure 2). It shown that 10 and 20 t ha^−1^ biochar enhanced the proportion of open PSII reaction centers and photosynthetic electron transfer rates in peanut leaves and reduced heat dissipation, which enable full use of the light energy absorbed in leaves for photosynthesis, and increased peanut yield. Our results are in agreement with those of Ali et al. (2020), who reported that appropriate rate of biochar increased qP and decreased NPQ at maturity stage. Biochar application improved nitrogen uptake from the soil (Sadaf et al., 2017), and a higher nitrogen concentration increased Φ_*PSII*_, qP and decreased NPQ (Lin et al., 2013). Additionally, it’s probably because biochar application enhanced leaf chlorophyll content (Feng et al., 2021), which ensured the synthesis of various enzymes and electron transporter in photosynthetic carbon assimilation, and consequently ameliorate photosynthetic function in leaves (Hou et al., 2021). Thus, the light energy absorbed by leaf was more used in photochemical processes, which led to the increase of qP and decrease of NPQ. In Summary, these results confirmed the potential of biochar for improving chlorophyll fluorescence traits. The internal mechanisms for biochar improving chlorophyll fluorescence traits merit further investigation.

### Effect of Biochar on Peanut Yield

Significant differences of pod yield were observed at least 20–40 t ha^−1^ biochar application in pot experiment (Xu et al., 2015). In our study, 10 t ha^−1^ biochar produced the maximum peanut yield (and kernel yield and shelling percentage; Table 4), as reported by Ye et al. (2019). Yamato et al. (2006) reported that 10 t ha^−1^ biochar application combined with fertilizer in infertile soil increased peanut yield by 50%. Similarly, the biochar application rate of 10 t ha^−1^ significantly increased peanut pod yield by 23% compared to the inorganic fertilizer only treatment (Agegnehu et al., 2015). In another study, rice husk and cottonseed husk biochar applications at 50 t ha^−1^ increased peanut yields by 16.8 and 14.4%, respectively, relative to the no-biochar amendment treatment (Tan et al., 2018). In this study, B40 decreased peanut yield, relative to B10 (Table 4). Some studies have reported that high rates of biochar can cause nitrogen immobilization and decrease nitrogen accumulation due to the high C/N ratio, reducing yield (Lehmann et al., 2002; Asai et al., 2009; Li et al., 2018; Yan et al., 2019). Despite the variation between studies, legumes generally respond better to biochar than other crops. For example, biochar application increased the yields of legumes, wheat, maize, and rice by about 30, 11, 8, and 7%, respectively, Liu et al. (2013). Biochar has strong potential to improve crop productivity, especially in drought and poor soils (Batool et al., 2015; Haider et al., 2017; Hussain et al., 2017). The large interannual variability in rainfall is the main climatic factor during pod formation period, causing fluctuations in peanut yield (Craufurd et al., 2006). High soil moisture content is conducive to pod filling in peanut. In our study, August 2019 had more rainfall than August 2018 (Figure 1), and the peanut yields differed accordingly.

The pod setting stage is critical for peanut yield formation. In our study, B10 significantly improved the photosynthetic capacity of peanut at pod set (Figure 4), ensuring reproductive growth during the critical growth period and increasing peanut yield. The regression coefficient between P_*n*_ and peanut yield was higher at pod set than at flowering in both years (Figure 4), indicating that photosynthetic capacity at pod set had a positive effect on yield. Overall, the increased yield at 10 t ha^−1^ biochar might be due to an enhanced photosynthetic capacity of functional leaves (Figure 5).

## Conclusion

Biochar application had a significant positive effect on photosynthetic capacity and yield in peanut. Maximum photochemical efficiency, actual photochemical efficiency, photochemical quenching, gas exchange parameters, leaf nitrogen content, plant nitrogen accumulation, yield, and yield components of peanut with increasing biochar application rate to 10 t ha^−1^ (B10). B10 significantly enhanced Φ_*PSII*_ and qP in functional leaves of peanut due to the transfer of more absorbed energy to photochemical reactions, ensuring a higher photosynthetic capacity at flowering and pod set and higher peanut yield than the other biochar rates. These results are in agreement with our hypothesis. Therefore, 10 t ha^−1^ biochar is recommended for increasing peanut yield in Northwest Liaoning, China. The results from this study enhances our understanding of the effects of biochar application on peanut photosynthesis and yield.

## Data Availability Statement

The original contributions presented in the study are included in the article/supplementary material, further inquiries can be directed to the corresponding author/s.

## Author Contributions

GX, TW, DC, and TC designed the experiment. SW, YW, and QY conducted the experiments, collected and analyzed the data, and prepared the manuscript. JZ, YC, and KS revised the manuscript. All authors contributed to the article and approved the submitted version.

## Conflict of Interest

The authors declare that the research was conducted in the absence of any commercial or financial relationships that could be construed as a potential conflict of interest.

## References

[B1] Abbas T. Rizwan M. Ali S. Adrees M. Zia-ur-Rehman M. Qayyum M. F. (2017). Effect of biochar on alleviation of cadmium toxicity in wheat (*Triticum aestivum* L.) grown on Cd-contaminated saline soil. *Environ. Sci. Pollut. Res.* 25 25668–25680. 10.1007/s11356-017-8987-4 28397121

[B2] Abideen Z. Koyro H. W. Huchzermeyer B. Gul B. Khan M. A. (2020). Impact of a biochar or a biochar-compost mixture on water relation, nutrient uptake and photosynthesis of *Phragmites karka*. *Pedosphere* 30 466–477. 10.1016/S1002-0160(17)60362-X

[B3] Agegnehu G. Bass A. M. Nelson P. N. Muirhead B. Wright G. Bird M. I. (2015). Biochar and biochar-compost as soil amendments: effects on peanut yield, soil properties and greenhouse gas emissions in tropical North Queensland, Australia. *Agric. Ecosys. Environ.* 213 72–85. 10.1016/j.agee.2015.07.027

[B4] Ali I. Ullah S. He L. Zhao Q. Iqbal A. Wei S. Q. (2020). Combined application of biochar and nitrogen fertilizer improves rice yield, microbial activity and N-metabolism in a pot experiment. *Peer J.* 8:e10311. 10.7717/peerj.10311 33240639 PMC7668215

[B5] Ali S. Xu Y. Jia Q. Ma X. Ahmad I. Adnan M. (2018). Interactive effects of plastic film mulching with supplemental irrigation on winter wheat photosynthesis, chlorophyll fluorescence and yield under simulated precipitation conditions. *Agri. Water Manag.* 207 1–14. 10.1016/j.agwat.2018.05.013

[B6] Asai H. Samson B. K. Stephan H. M. Songyikhangsuthor K. Homma K. Kiyono Y. (2009). Biochar amendment techniques for upland rice production in Northern Laos 1. Soil physical properties, leaf SPAD and grain yield. *Field Crops Res.* 111 81–84. 10.1016/j.fcr.2008.10.008

[B7] Bai W. Sun Z. Zheng J. Hou Z. Liu Y. Feng L. (2014). Effect of different planting patterns on maize growth and yield in western Liaoning province. *Acta Agron. Sinica.* 40 181–189. 10.3724/SP.J.1006.2014.00181

[B8] Baker N. R. (2008). Chlorophyll fluorescence: a probe of photosynthesis in vivo. *Ann. Rev. Plant Biol.* 59 89–113. 10.1146/annurev.arplant.59.032607.092759 18444897

[B9] Batool A. Taj S. Rashid A. Khalid A. Qadeer S. Saleem A. R. (2015). Potential of soil amendments (biochar and gypsum) in increasing water use efficiency of Abelmoschus esculentus L. Moench. *Front. Plant Sci.* 6:733. 10.3389/fpls.2015.00733 26442046 PMC4566053

[B10] Bruun E. W. Petersen C. T. Hansen E. Holm J. K. Hauggaard-Nielsen H. (2014). Biochar amendment to coarse sandy subsoil improves root growth and increases water retention. *Soil Use Manage.* 30 109–118. 10.1111/sum.12102

[B11] Chen Y. S. Wang Z. J. Shen Z. J. Ou Z. L. Xu D. C. Yuan Z. X. (2017). Effects of oxytetracycline on growth and chlorophyll fluorescence in rape (*Brassica campestris* L.). *Pol. J. Environ. Stud.* 26 995–1001. 10.15244/pjoes/67575

[B12] Clough T. J. Condron L. M. Kammann C. Muller C. (2013). A review of biochar and soil nitrogen dynamics. *Agronomy* 3 275–293. 10.3390/agronomy3020275

[B13] Craufurd P. Q. Prasad P. V. V. Waliyar F. Taheri A. (2006). Drought, pod yield, pre-harvest Aspergillus infection and aflatoxin contamination on peanut in Niger. *Field Crops Res.* 98 20–29. 10.1016/j.fcr.2005.12.001

[B14] El-Naggar A. Lee S. S. Rinklebe J. Farooq M. Song H. Sarmah A. K. (2019). Biochar application to low fertility soils: a review of current status, and future prospects. *Geoderma* 337 536–554. 10.1016/j.geoderma.2018.09.034

[B15] Evans J. R. (1989). Photosynthesis and nitrogen relationships in leaves of C3 plants. *Oecologia* 78 9–19. 10.1007/bf00377192 28311896

[B16] Fang X. Li Y. Nie J. Wang C. Huang K. Zhang Y. K. (2018). Effects of nitrogen fertilizer and planting density on the leaf photosynthetic characteristics, agronomic traits and grain yield in common buckwheat (*Fagopyrum esculentum* M.). *Field Crops Res.* 219 160–168. 10.1016/j.fcr.2018.02.001

[B17] FAOSTAT (2018). *Food Agriculture and Organization of the United Nations (FAO).* Available online at: http://www.fao.org/faostat/en/#data/QC (accessed 8 Aug, 2020).

[B18] Feng W. Y. Yang F. Cen R. Liu J. Qu Z. Y. Miao Q. F. (2021). Effects of straw biochar application on soil temperature, available nitrogen and growth of corn. *J. Environ. Manage.* 277:111331. 10.1016/j.jenvman.2020.111331 32949951

[B19] Guo L. L. Bornø M. L. Niu W. Q. Liu F. L. (2021). Biochar amendment improves shoot biomass of tomato seedlings and sustains water relations and leaf gas exchange rates under different irrigation and nitrogen regimes. *Agri. Water Manag.* 245:106580. 10.1016/j.agwat.2020.106580

[B20] Haider G. Steffens D. Moser G. Müller C. Kammann C. I. (2017). Biochar reduced nitrate leaching and improved soil moisture content without yield improvements in a four-year field study. *Agric. Ecosyst. Environ.* 237 80–94. 10.1016/j.agee.2016.12.019

[B21] Hazrati S. Tahmasebi S. Z. Modarres S. S. A. M. Mokhtassi B. A. Nicola S. (2016). Effects of water stress and light intensity on chlorophyll fluorescence parameters and pigments of Aloe vera L. *Plant Physiol. Biochem.* 106 141–148. 10.1016/j.plaphy.2016.04.046 27161580

[B22] He Y. H. Yao Y. X. Ji Y. H. Deng J. Zhou G. Y. Liu R. Q. (2020). Biochar amendment boosts photosynthesis and biomass in C3 but not C4 plants: a global synthesis. *GCB Bioenergy* 12 605–617. 10.1111/gcbb.12720

[B23] Hou W. H. Zhang Y. X. Wang H. J. Zhang Q. X. Hou M. L. Cong B. M. (2021). Effects of nitrogen application level on leaf photosynthetic characteristics and chlorophyll fluorescence characteristics of *Leymus chinensis*. *Acta Agrestia Sinica* 29 532–536. 10.11733/j.issn.1007-0435.2021.03.014

[B24] Huang X. F. Li S. Q. Li S. Y. Ye G. Y. Lu L. J. Zhang L. (2019). The effects of biochar and dredged sediments on soil structure and fertility promote the growth, photosynthetic and rhizosphere microbial diversity of *Phragmites communis* (Cav.) Trin. ex Steud. *Sci. Total Environ.* 697:134073. 10.1016/j.scitotenv.2019.134073 31473547

[B25] Hussain M. Farooq M. Nawaz A. Al-Sadi A. M. Solaiman Z. M. Alghamdi S. S. (2017). Biochar for crop production: potential benefits and risks. *J. Soil Sediment.* 17 685–716. 10.1007/s11368-016-1360-2

[B26] Ippolito J. A. Cui L. Q. Kammann C. Mönnig N. W. Estavillo J. M. Mendizabal T. F. (2020). Feedstock choice, pyrolysis temperature and type influence biochar characteristics: a comprehensive meta-data analysis review. *Biochar* 2 421–438. 10.1007/s42773-020-00067-x

[B27] Kammann C. I. Linsel S. Gößling J. W. Koyro H. W. (2011). Influence of biochar on drought tolerance of *Chenopodium quinoa* Willd and on soil-plant relations. *Plant Soil* 345 195–210. 10.1007/s11104-011-0771-5

[B28] Kassambara A. (2015). *Factoextra: Visualization of the Outputs of a Multivariate Analysis. R Package Version 1: Statistical Tools for Highthroughput Data Analysis.* Available online at: http://www.sthda.com

[B29] Klughammer C. Schreiber U. (2008). Complementary PS II quantum yields calculated from simple fluorescence parameters measured by PAM fluorometry and the saturation pulse method. *PAM Appl. Notes* 1 27–35.

[B30] Kooten O. V. Snel J. F. H. (1990). The use of chlorophyll fluorescence nomenclature in plant stress physiology. *Photosynth. Res.* 25 147–150. 10.1007/BF00033156 24420345

[B31] Kramer D. M. Johnson G. Kiirats O. Edwards G. E. (2004). New fluorescence parameters for the determination of QA redox state and excitation energy fluxes. *Photosynth. Res.* 79 209–218. 10.1023/B:PRES.0000015391.99477.0d 16228395

[B32] Laghari M. Mirjat M. S. Hu Z. Fazal S. Xiao B. Hu M. (2015). Effects of biochar application rate on sandy desert soil properties and sorghum growth. *Catena* 135 313–320. 10.1016/j.catena.2015.08.013

[B33] Lehmann J. da Silva J. P. Jr. Steiner C. Nehls T. Zech W. Glaser B. (2002). Nutrient availability and leaching in an archaeological Anthrosol and a Ferralsol of the Central Amazon basin: fertilizer, manure and charcoal amendments. *Plant Soil* 249 343–357. 10.1023/A:1022833116184

[B34] Lehmann J. Rillig M. C. Thies J. Masiello C. A. Hockaday W. C. Crowley D. (2011). Biochar effects on soil biota-a review. *Soil Biol. Biochem.* 43 1812–1836. 10.1016/j.soilbio.2011.04.022

[B35] Leng L. J. Xu S. Y. Liu R. F. Yu T. Zhou X. M. Leng S. Q. (2020). Nitrogen containing functional groups of biochar: an overview. *Bioresour. Technol.* 298:122286. 10.1016/j.biortech.2019.122286 31690478

[B36] Li G. Wan S. Zhou J. Yang Z. Qin P. (2010). Leaf chlorophyll fluorescence, hyperspectral reflectance, pigments content, malondialdehyde and proline accumulation responses of castor bean (*Ricinus communis* L.) seedlings to salt stress levels. *Ind. Crops Prod.* 31 13–19. 10.1016/j.indcrop.2009.07.015

[B37] Li S. L. Zhou P. Shang G. (2018). Positive effects of apple branch biochar on wheat yield only appear at a low application rate, regardless of nitrogen and water conditions. *J. Soil Sediment.* 18 3235–3243. 10.1007/s11368-018-1994-3

[B38] Li X. Y. Gong J. D. (2002). Effects of different ridge: furrow ratios and supplemental irrigation on crop production in ridge and furrow rainfall harvesting system with mulches. *Agri Water Manag.* 54 243–254. 10.1016/S0378-3774(01)00172-X

[B39] Li Y. S. Wu L. H. Zhao L. M. Lu X. H. Fan Q. L. Zhang F. S. (2007). Influence of continuous plastic film mulching on yield, water use efficiency and soil properties of rice fields under nonflooding condition. *Soil Tillage Res.* 93 370–378. 10.1016/j.still.2006.05.010

[B40] Lin Y. C. Hu Y. G. Ren C. Z. Guo L. C. Wang C. L. Jiang Y. (2013). Effects of nitrogen application on chlorophyll fluorescence parameters and leaf gas exchange in naked oat. *J. Integr. Agric.* 12 2164–2171. 10.1016/S2095-3119(13)60346-9

[B41] Lin Z. Liu Q. Liu G. Cowie A. L. Bei Q. Liu B. I. U. (2017). Effects of different biochars on *Pinus elliottii* growth, N use efficiency, soil N2O and CH4 emissions and C storage in a subtropical area of China. *Pedosphere* 27 248–261. 10.1016/S1002-0160(17)60314-X

[B42] Liu Q. Zhang Y. H. Liu B. J. Amonette J. E. Lin Z. B. Liu G. (2018). How does biochar influence soil N cycle? A meta-analysis. *Plant Soil* 426 211–225. 10.1007/s11104-018-3619-4

[B43] Liu X. Y. Zhang A. F. Ji C. Y. Joseph S. Bian R. J. Li L. Q. (2013). Biochar’s effect on crop productivity and the dependence on experimental conditions—a meta-analysis of literature data. *Plant Soil* 373 583–594. 10.1007/s11104-013-1806-X

[B44] Liu Z. X. Gao F. Yang J. Q. Zhen X. Y. Li Y. Zhao J. H. (2019). Photosynthetic characteristics and uptake and translocation of nitrogen in peanut in a wheat-peanut rotation system under different fertilizer management regimes. *Front. Plant Sci.* 10:86. 10.3389/fpls.2019.000086 PMC6374608 30792727

[B45] Long J. R. Ma G. H. Wan Y. Z. Song C. F. Sun J. Qin R. J. (2013). Effects of nitrogen fertilizer level on chlorophyll fluorescence characteristics in flag leaf of super hybrid rice at late growth stage. *Rice Sci.* 20 220–228. 10.1016/S1672-6308(13)60138-9

[B46] Luo H. Y. Xu Z. J. Li Z. D. Li X. P. Lv J. W. Ren X. P. (2017). Development of SSR markers and identification of major quantitative trait loci controlling shelling percentage in cultivated peanut (*Arachis hypogaea* L.). *Theor. Appl. Genet.* 130 1635–1648. 10.1007/s00122-017-2915-3 28508097 PMC5511596

[B47] Marks E. A. N. Mattana S. Alcañiz J. M. Pérez-Herrero E. Domene X. (2016). Gasifier biochar effects on nutrient availability, organic matter mineralization, and soil fauna activity in a multi-year Mediterranean trial. *Agric. Ecosyst. Environ.* 215 30–39. 10.1016/j.agee.2015.09.004

[B48] Maxwell K. Johnson G. N. (2000). Chlorophyll fluorescence-a practical guide. *J. Exp. Bot.* 51 659–668. 10.1093/jexbot/51.345.659 10938857

[B49] Nguyen T. T. N. Wallace H. M. Xu C. Y. Xu Z. H. Farrar M. B. Joseph S. (2017). Short-term effects of organo-mineral biochar and organic fertilisers on nitrogen cycling, plant photosynthesis, and nitrogen use efficiency. *J. Soil. Sediment.* 17 2763–2774. 10.1007/s11368-017-1839-5

[B50] Novak J. M. Busscher W. J. Watts D. W. Amonette J. E. Ippolito J. A. Lima I. M. (2012). Biochars impact on soil-moisture storage in an ultisol and two aridisols. *Soil Sci.* 177 310–320. 10.1097/SS.0b013e31824e5593

[B51] Peel M. C. Finlayson B. L. McMahon T. A. (2007). Updated world map of the Köppen Geiger climate classification. *Hydrol. Earth Syst. Sci. Discuss.* 11 1633–1644. 10.5194/hess-11-1633-2007

[B52] Perkins R. Williamson C. Lavaud J. Mouget J. L. Campbell D. A. (2018). Time-dependent upregulation of electron transport with concomitant induction of regulated excitation dissipation in Haslea diatoms. *Photosynth. Res.* 137 377–388. 10.1007/s11120-018-0508-X 29663190 PMC6182385

[B53] Rehman M. Liu L. J. Bashir S. Saleema M. H. Chen C. Peng D. X. (2019). Influence of rice straw biochar on growth, antioxidant capacity and copper uptake in ramie (*Boehmeria nivea* L.) grown as forage in aged copper contaminated soil. *Plant Physiol. Biochem.* 138 121–129. 10.1016/j.plaphy.2019.02.021 30861402

[B54] Rehman M. Z. Rizwan M. Ali S. Fatima N. Yousaf B. Naeem A. (2016). Contrasting effects of biochar, compost and farm manure on alleviation of nickel toxicity in maize (*Zea mays* L.) in relation to plant growth, photosynthesis and metal uptake. *Ecotoxicol. Environ. Saf.* 133 218–225. 10.1016/j.ecoenv.2016.07.023 27467022

[B55] Sadaf J. Shah G. A. Shahzad K. Ali N. Shahid M. Ali S. (2017). Improvements in wheat productivity and soil quality can accomplish by co-application of biochars and chemical fertilizers. *Sci. Total Environ.* 607–608 715–724. 10.1016/j.scitotenv.2017.06.178 28711001

[B56] Steinmetz Z. Wollmann C. Schaefer M. Buchmann C. David J. Tröger J. (2016). Plastic mulching in agriculture. Trading short-term agronomic benefits for long-term soil degradation? *Sci. Total Environ.* 550 690–705. 10.1016/j.scitotenv.2016.01.153 26849333

[B57] Sun T. Li G. Ning T. Y. Zhang Z. M. Mi Q. H. Lal R. (2018). Suitability of mulching with biodegradable film to moderate soil temperature and moisture and to increase photosynthesis and yield in peanut. *Agri Water Manag.* 208 214–223. 10.1016/j.agwat.2018.06.027

[B58] Tan G. C. Wang H. Y. Xu N. Liu H. B. Zhai L. M. (2018). Biochar amendment with fertilizers increases peanut N uptake, alleviates soil N2O emissions without affecting NH3 volatilization in field experiments. *Environ. Sci. Pollut. Res.* 25 8817–8826. 10.1007/s11356-017-1116-6 29327196

[B59] Tang J. W. Zhang S. D. Zhang X. T. Chen J. H. He X. Y. Zhang Q. Z. (2020). Effects of pyrolysis temperature on soil-plant-microbe responses to *Solidago canadensis* L.-derived biochar in coastal saline-alkali soil. *Sci. Total Environ.* 731:138938. 10.1016/j.scitotenv.2020.138938 32408208

[B60] Xia G. M. Wang Y. J. Hu J. Q. Wang S. J. Zhang Y. Wu Q. (2021a). Effects of supplemental irrigation on water and nitrogen use, yield, and kernel quality of peanut under nitrogen-supplied conditions. *Agri Water Manag.* 243:106518. 10.1016/j.agwat.2020.106518

[B61] Xia G. M. Wang Y. J. Wang S. J. Yang Q. F. Chi D. C. (2021b). Effects of irrigation methods and biochar on peanut root, phosphorus utilization and yield. *T. Chin. Soc. Agric. Mach.*

[B62] Xiang Y. Z. Deng Q. Duan H. L. Guo Y. (2017). Effects of biochar application on root traits: a meta-analysis. *GCB Bioenergy* 9 1563–1572. 10.1111/gcbb.12449

[B63] Xu C. Y. Hosseini B. S. Hao Y. Rachaputi R. C. N. Wang H. Xu Z. H. (2015). Effect of biochar amendment on yield and photosynthesis of peanut on two types of soils. *Environ. Sci. Pollut. Res.* 22 6112–6125. 10.1007/s11356-014-3820-9 25395326

[B64] Yamato M. Okimori Y. Wibowo I. F. Anshori S. Ogawa M. (2006). Effects of the application of charred bark of Acacia mangium on the yield of maize, cowpea and peanut, and soil chemical properties in South Sumatra, Indonesia. *Soil Sci. Plant Nutr.* 52 489–495. 10.1111/j.1747-0765.2006.00065.x

[B65] Yan Q. Y. Dong F. Li J. H. Duan Z. Q. Yang F. Li X. (2019). Effects of maize straw-derived biochar application on soil temperature, water conditions and growth of winter wheat. *Eur. J. Soil Sci.* 70 1280–1289. 10.1111/ejss.12863

[B66] Ye L. L. Camps A. M. Shen Q. H. Lehmann J. Singh B. Sabir M. (2019). Biochar effects on crop yields with and without fertilizer: a meta-analysis of field studies using separate controls. *Soil Use Manage.* 36 2–18. 10.1111/sum.12546

[B67] Ye Z. X. Liu L. Y. Tan Z. X. Zhang L. M. Huang Q. Y. (2020). Effects of pyrolysis conditions on migration and distribution of biochar nitrogen in the soil-plant-atmosphere system. *Sci. Total Environ.* 723:138006. 10.1016/j.scitotenv.2020.138006 32222503

[B68] Zelitch I. (1982). The close relationship between net photosynthesis and crop yield. *BioScience* 32 796–802. 10.2307/1308973

